# Matrix-Guided Vascular-like Cord Formation by MRC-5 Lung Fibroblasts: Evidence of Structural and Transcriptional Plasticity

**DOI:** 10.3390/cells14191519

**Published:** 2025-09-29

**Authors:** Nikoleta F. Theodoroula, Alexandros Giannopoulos-Dimitriou, Aikaterini Saiti, Aliki Papadimitriou-Tsantarliotou, Androulla N. Miliotou, Giannis Vatsellas, Yiannis Sarigiannis, Eleftheria Galatou, Christos Petrou, Dimitrios G. Fatouros, Ioannis S. Vizirianakis

**Affiliations:** 1 Laboratory of Pharmacology, School of Pharmacy, Aristotle University of Thessaloniki, 54124 Thessaloniki, Greece; nikol_theodor@hotmail.com (N.F.T.); gianalex@auth.gr (A.G.-D.); skaikater@auth.gr (A.S.); alikipapadi@pharm.auth.gr (A.P.-T.); 2 Department Health Sciences, School of Life & Health Sciences, University of Nicosia, 2417 Nicosia, Cyprus; miliotou.a@unic.ac.cy (A.N.M.); sarigiannis.i@unic.ac.cy (Y.S.); galatou.e@unic.ac.cy (E.G.); petrou.c@unic.ac.cy (C.P.); 3 Greek Genome Center, Biomedical Research Foundation Academy of Athens, 11527 Athens, Greece; gvatsellas@bioacademy.gr; 4 Department of Pharmaceutical Technology, School of Pharmacy, Aristotle University of Thessaloniki, 54124 Thessaloniki, Greece; dfatouro@pharm.auth.gr

**Keywords:** fibroblast plasticity, tissue engineering, Matrigel, transcriptomic remodeling, cord-like formation

## Abstract

The role of mesenchymal-to-endothelial transition in the angiogenic response remains controversial. In this study, we investigated whether human fetal lung fibroblasts (MRC-5 cells) exhibit morphological plasticity in a biomimetic extracellular matrix environment. To this end, MRC-5 cells were first cultured on and within Matrigel hydrogel and then studied with tube formation assays, confocal/fluorescence microscopy, invasion assays, and transcriptomic profiling. In addition, quantitative assessment for cord formation and gene expression was conducted via qPCR and RNA sequencing. In this study, MRC-5 cells quickly self-organized into cord-like networks, resembling early stages of vascular patterning, and at higher densities, invaded the hydrogel and formed spheroid-like aggregates. Transcriptomic analysis revealed upregulation of genes related to nervous system development and synaptic signaling in Matrigel-grown MRC-5 cultures. Collectively, these findings suggest that MRC-5 fibroblasts display structural and transcriptional plasticity in 3D Matrigel cultures, forming vascular-like cords that are more likely to resemble early developmental morphologies or neuroectodermal-like transcriptional signatures than definitive endothelial structures. This work underscores the potential of fibroblasts as an alternative cell source for vascular tissue engineering and highlights a strategy to overcome current limitations in autologous endothelial cell availability for regenerative applications.

## 1. Introduction

The formation of new blood vessels by endothelial cells (ECs) is of great importance to clinicians and researchers as it has significant potential to make a major impact on the treatment of cardiovascular disease [1]. In the lungs, ECs participate in alveolar development and repair through a variety of interactions within the vascular niche, generating angiocrine signals that modulate the behavior of epithelial and mesenchymal components [2] (Mammoto & Mammoto, 2019). Tissue engineering has emerged as a promising approach to the creation of a bio-compatible vessel graft with the ability to grow, remodel, and repair in vivo [3]. Considerable advancements in the area of vessel engineering have been reported [4], since Weinberg and Bell, in 1986, succeeded in creating—for the first time—a blood vessel from collagen, endothelial cells, smooth muscle cells, and fibroblasts [5]. However, after a few decades of research, despite considerable progress in the field of vascular tissue engineering, numerous difficulties still remain. The major issues include considerable technical obstacles to acquiring autologous ECs for injury treatment and tissue engineering due to the limited source, harvest procedure, and proliferative capacity of these cells [6,7,8,9]. However, some of these difficulties in vascular tissue engineering can be overcome using endothelial progenitor cells (EPCs) [10,11]. This subtype of stem cells has the potent capacity to proliferate in vitro and differentiate into mature ECs [12]. In addition, there are multipotent stromal cells (MSCs) that can differentiate into a variety of lineages [13]. However, the clinical use of MSCs and EPCs has also been limited due to difficulties associated with their harvest, culture, and expansion [14,15,16]. Thus, the demand for an alternative cell source to develop a suitable method for obtaining abundant human ECs in vitro, for the formation of new blood vessels, continues to grow.

Fibroblasts are traditionally defined as diverse mesenchymal cells that participate in tissue homeostasis and disease [17]. MRC-5 cells are well-known to express core features of mesenchymal stem cells (MSCs), such as the ability to differentiate along multiple lineages, propagate immunomodulation, and express MSC surface markers, raising the possibility that these cells may exert some plasticity beyond being restricted to a fibroblastic identity [18]. They are involved in the formation, maintenance, and degradation of the extracellular matrix (ECM), thereby contributing to the mechanical properties of tissues [19,20]. It is therefore not surprising that fibroblasts have been suggested to contribute to angiogenesis through modulation of the ECM network and protein secretion, crucial for lumen formation and EC sprouting [21]. These angiogenic mediators include vascular endothelial growth factor [22] and angiogenic chemokines. Whether fibroblasts directly contribute to angiogenesis by converting to endothelial cells through mesenchymal to endothelial transition (MEndoT) needs to be further investigated [23]. Transdifferentiation of fibroblasts to ECs may provide a novel therapeutic avenue for cardiovascular diseases [24]. Recently, several studies have demonstrated that fibroblasts may be reprogrammed into ECs in vitro [25,26,27,28,29,30,31].

The molecular underpinnings of cellular plasticity and transdifferentiation have lately been addressed by transcriptomic profiling quantification [32]. Combining high-throughput expression analysis with functional enrichment methods, such as Gene Ontology (GO) and Reactome pathway enrichment, systematically identifies biological processes and signaling pathways that are communicatively activated during transitions of cell fate [33]. These methods offer important mechanistic information in order to discern true lineage reprogramming events from novice and transient phenotypic responses. One additional layer of validation, while also possibly complicating the accuracy of gene expression, is to use differential gene expression and enrichment analyses—especially for models based on a complex extracellular matrix (such as Matrigel)—where the morphological changes in a structure may not truly reflect the substantial underlying molecular changes taking place within a fibroblast-to-endothelial conversion model [34,35].

**Figure 1:**
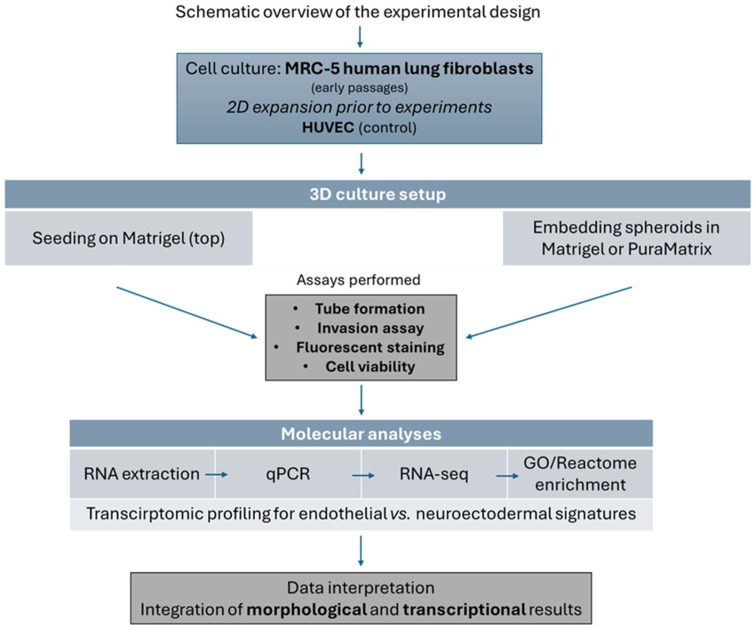
A schematic overview of the experimental design. The human MRC-5 lung fibroblasts were expanded under standard 2D culture conditions and then transferred to multidimensional extracellular matrix environments, whereas HUVEC cells served as control endothelial cells. Cells were seeded on top of Matrigel for tube formation assays or embedded into Matrigel/PuraMatrix for invasion assays. Functional assays comprised tube formation (Matrigel assay, ImageJ (Version 1.53t) quantification), invasion (Oris system, WimScratch analysis), fluorescent staining (DAPI for nuclei, PI for dead cells), and cell viability (CCK-8, dead staining). Molecular analyses involved RNA extraction, qPCR, RNA-seq, and GO/Reactome enrichment analyses. Data interpretation was founded on the conjoint analysis of morphological and transcriptional data.

In this context, our study aimed to examine the morphological and transcriptional responses of MRC-5 lung fibroblasts presented in a three-dimensional (3D) extracellular matrix environment, as shown in Figure 1. Utilizing Matrigel to culture these cells, we explored whether the matrix microenvironment would impose phenotypic features that are associated with cells performing in some form of vascular organization. Building a point of distinction between these structures from true endothelial or epithelial constructs, we utilized a reference endothelial cell model and performed transcriptomic profiling. Furthermore, because the MRC-5 fibroblasts originate from a lung source, we considered the possibility of lineage acquisition that was characteristic of cells related to the lung, such as neuroectodermal-like transcriptional signatures. Our aim was to define the extent and nature of fibroblast plasticity as it pertains to the interactions within a biomimetic matrix environment, with implications for regenerative biology, tissue engineering, and disease modeling.

## 2. Materials and Methods

### 2.1. Hydrogel

Matrigel hydrogel was used to study the phenotypes of human lung fibroblast MRC-5 cells grown on top of the matrices or embedded. Matrigel™ (Corning, New York, NY, USA) consists of basal membrane extract derived from Engelbreth–Holm–Swarm mouse sarcoma cells. At room temperature, it solidifies and forms a hydrogel. The extracellular matrix components present in this hydrogel include laminin, collagen IV, entactin, nidogen, and heparan sulfate proteoglycans. Growth factors such as TGF-β, basic fibroblast growth factor, insulin-like growth factor-1, and tissue plasminogen activator are also present. Matrigel has previously been used and has been shown to stimulate cells in vitro.

### 2.2. Cell Cultures and Treatments

The already established human normal MRC-5 (fetal lung fibroblasts) are stored and routinely used in our laboratory. The MRC-5 cells were grown in culture (37 °C; humidified atmosphere containing 5% *v*/*v* CO_2_) in 25 cm^2^ flasks and/or in a 6- or 96-multiwell microplate, according to the type of assay to be performed, in 1× Dulbecco’s modification of Eagle’s medium (DMEM, Corning) supplemented with 10% (*v*/*v*) fetal bovine serum (FBS) and 1% penicillin/streptomycin (Gibco, Waltham, MA, USA). The treatments were performed at sub-confluence, and then the cells were detached by using trypsin-EDTA (0.25% *w*/*v*, Life Technologies, Carlsbad, CA, USA) [36].

The human umbilical vein endothelial cell line (HUVEC cells) was grown in Endothelial Cell Culture Medium (Sigma-Aldrich, Merck KGaA, Darmstadt, Germany), as previously published [37].

### 2.3. Tube Formation Assay

The in vitro tube formation assay on Corning^®^ Matrigel™ Matrix (Corning, New York, NY, USA) was conducted according to a previously published protocol [38]. Following slow overnight thawing on the ice at 4 °C of Corning Matrigel Matrix, according to the recommendations provided in the product’s Guidelines for Use, 250 μL of Matrigel solution was dispensed into each well of a 24-well plate and incubated for 30 min at 37 °C, for the experiment of MRC-5 and HUVEC cells grown on Matrigel. Cell suspensions were prepared by trypsinizing the cell monolayers with trypsin–EDTA and resuspending the cells in culture medium of 10% FBS. A total of 500 μL of cells was seeded on top of 10 mg/mL Matrigel at a starting density of 10^5^ cells/mL, 1.5 × 10^5^ cells/mL, or 3 × 10^5^ cells/mL, at 37 °C. The matrix-coated well plates were incubated for 24 h for cell tube formation. After being incubated for 24 h at 37 °C, images of tube formation were taken accordingly. Additional experiments were conducted to determine the optimal concentration, which allowed cells to show either an increase or a decrease in tube formation ability. For experiments on tube formation, samples were photographed with magnifications of 20× and 40× using an inverted light microscope. Each experiment was repeated at least three times independently.

### 2.4. Quantitative ImageJ Analysis for Angiogenic Assay

The ImageJ software (Version 1.53t, Wayne Rasband, National Institutes of Health, Bethesda, MD, USA) was used by applying the “Angiogenesis Analyzer” plug-in tool to analyze several different parameters related to tube formation as described in Section 3. Indicators of the analyzed tube-forming elements included the number of junctions, segments, meshes, etc. It was also used to quantitatively evaluate the vessel-like network organization. “Segments” indicate the portions of an angiogenic capillary with two ends connected to two junction points. “Junctions” are the multi-intersection junctions with furcates of three or more branches in the angiogenic structures. “Meshes” represent closed areas formed by segments.

### 2.5. Fluorescent Staining

After 24 h of incubation, the cells were washed twice with PBS and fixed in Intracellular Staining Fixation Buffer (BioLegend, Cat. No. 420801) for 20 min. The cells were then rinsed with PBS and permeabilized with Intracellular Staining Permeabilization Wash Buffer 10× diluted to 1× in deionized (DI) water (BioLegend, Cat. No. 421002) for 10 min. The coverslips were mounted onto glass microscope slides in Prolong Gold Antifade Reagent with DAPI, sealed, and left overnight in the dark at 4 °C. The fluorescent signals of (DAPI)-labeled cell nuclei were imaged with a 40× oil-immersion lens under a Zeiss LSM 780 CLSM (Carl Zeiss Microscopy GmbH, Berlin, Germany) using appropriate filters.

### 2.6. Cell Invasion Assay

The Oris Universal Cell Migration Assembly kit was purchased from Platypus Technologies, LLC (Fitchburg, WI, USA). Each 96-well plate of the kit contained Cell Seeding Stoppers (2 mm in diameter) for creating a detection zone at the center of each well. The wells of the 96-well plate were coated with 50 µL/well of diluted Matrigel^®^ (100 µg/mL). The plate was placed in a 37 °C incubator with 5% CO_2_ to allow the biomatrix material to polymerize for 2 h to study MRC-5 cell invasion. After 2 h, the plate was removed from 37 °C, and Cell Seeding Stoppers were placed. One well without a Cell Seeding Stopper served as the positive control. MRC-5 cells (100 µL/well, 100,000–400,000 cells/mL stock) were seeded into each well of the plate. The seeded plate containing the Oris™ Cell Seeding Stoppers was incubated in a humidified chamber (37 °C, 5% CO_2_) overnight to permit cell attachment. After the cells were attached to the matrices, the spacers were removed. Media were removed and wells were washed with 100 μL of sterile PBS. After washing, 100 µL of culture media was added, and the cell plate was cooled to 4 °C for 5 min. The top layer of the matrix was prepared on ice by diluting Matrigel^®^, 4–8 mg/mL. Then, 50 µL of the Matrigel^®^ top layer was added. The addition of the two Matrigel™ layers ensured that the assay measures invasion, as opposed to 2D wound healing migration. The cell plate was then placed at 37 °C for the biomatrix top layer to polymerize. After 30 min, 100 µL/well of additional media was added to each well, and the plate was incubated in a humidified chamber (37 °C, 5% CO_2_) to permit cell invasion. Cells were examined microscopically after 24, 48, and 72 h to monitor the progression of invasion. Three independent biological experiments were performed for statistical analysis using Student’s *t*-test. The images obtained were quantified by WimScratch (Version 2022.2, Wimasis Image Analysis, Onimagin Technologies SCA, Córdoba, Spain).

### 2.7. In Vitro Cell Viability Assay

Cell Counting Kit-8 (CCK-8, Dojindo, Mashiki, Japan) was used to assess the viability of human lung fibroblasts both in a 2D monolayer dish culture and in 3D constructs on top of 10 mg/mL Matrigel matrix. MRC-5 cells were cultured in DMEM containing 1% penicillin/streptomycin solution and 10% FBS at 37 °C, under 5% CO_2_ conditions. Five independent biological experiments were performed for the measurement of cell viability, and all data presented are the average of triplicate experiments. After 48 h of incubation, the Cell Counting Kit-8 (CCK8, Sigma-Aldrich) reagent was added to each well, and the cells were incubated for 2 h at 37 °C. One hundred microliters of the supernatant were transferred into a 96-well plate, and the OD was assessed at 450 nm in a multifunction microplate reader. Wells containing only the CCK-8 reagent were used as a blank control.

Fluorescent dead staining was used to determine dead cells in the 3D cell-laden constructs according to the manufacturer’s instructions. Briefly, samples were gently washed in PBS three times. Propidium iodide (PI, 421301, BioLegend, San Diego, CA, USA) was used to stain dead cells (red) for 30 min in a cell incubator [39]. A fluorescence imaging system (EVOS FL imaging system, AMF4300, Life Technologies) was used for observation and image acquisition. The percentage of dead cells was calculated using Image-J software. Four random fields were counted for each sample.

### 2.8. RNA Extraction and Real-Time Polymerase Chain Reaction (qPCR)

MRC-5 cells were plated on top of Matrigel Matrix gel. After treatment for 48 h, total RNA was isolated from 3D cell cultures on top of 10 mg/mL Matrigel^®^ Matrix using the ReliaPrep™ miRNA Cell and Tissue Miniprep System (Z6211, Promega, Southampton, UK) according to the product protocol [40]. After isolation, RNA was tested qualitatively and quantitatively, respectively, via gel electrophoresis assay and a NanoDrop 2000 (Thermo Fisher Scientific, Waltham, MA, USA) spectrophotometer. RNA samples were subjected to reverse transcription using the PrimeScript™ RT-PCR Kit (RR014A, Takara Bio Inc., Tokyo, Japan), as per the manufacturer’s instructions. Quantitative RT-PCR analysis was performed using the KAPA SYBR FAST qPCR Kit (KK4602, Kapa Biosystems, Wilmington, MA, USA). The primer sequences of genes related to cell cycle and proliferation are presented in Supplementary Table S1. The primer sequences of the endothelial biomarker genes used in qPCR analysis are shown in Supplementary Table S2.

### 2.9. RNASeq, Gene Ontology, and Pathway Enrichment Analysis

MRC-5 cells were cultured under normal conditions, as described above, and on top of 10 mg/mL Matrigel at a starting density of 1.5 × 10^5^ cells/mL. Total RNA from 3 replicates of each condition was isolated with the RNeasy Kit (Qiagen, Limburg, The Netherlands), according to the manufacturer’s instructions. RNAseq libraries were prepared with the NEBNext Ultra II Directional RNA Library Prep Kit (New England Biolabs, Ipswich, MA, USA) for Illumina. Quality Control of the libraries was performed with the Agilent Bioanalyzer DNA1000 Kit (Agilent Technologies, Santa Clara, CA, USA) and Quantitation with the qubit HS spectrophotometric method. Approximately 25 million 100 bp Single-End reads were generated for each sample in the Illumina Novaseq 6000 system (Illumina Inc., San Diego, CA, USA).

Transcriptomic analyses were conducted using samples from two independent experiments, both performed under identical conditions with respect to Matrigel concentration and cell passage number. The primary modification between the two experiments was the duration of cell culture, allowing for the investigation of time-dependent transcriptomic alterations. In the first experiment, cells were cultured within Matrigel for 24 h, whereas in the second experiment, the culture duration was extended to 48 h. For the 48 h experiment, one of the three Matrigel replicates was removed from analysis due to low sequencing depth and outlier clustering in principal component analysis (PCA), leading to an analysis of two biological replicates per condition. The 24 h experiment retained all three biological replicates for each condition.

Differential expression analysis of RNAs identified in MRC-5 cells cultured in normal conditions compared to MRC-5 cells cultured in Matrigel using the bioinformatics tool DESeq2 (Version 1.49.4). The differentially expressed (DE) genes were determined based on a log2 fold change (FC) > 1 for upregulated genes and log2(FC) < −1 for downregulated genes and an adjusted *p* < 0.05, using the Wald test for hypothesis testing in DESeq2. Gene Ontology (GO) enrichment, as per molecular functions, biological processes, and cellular components, and Reactome pathway analysis were conducted via the cluster Profiler and Reactome PA packages, respectively, in R (https://www.r-project.org/).

### 2.10. Statistical Analysis

Data are presented as mean ± standard deviation (SD) of triplicate incubations. Student’s *t*-test and one-way analysis of variance (ANOVA) were employed to evaluate the data. The significance level was set at *p* ≤ 0.05.

## 3. Results

### 3.1. Optimal Cell Number for Tube Formation Assay

**Figure 2:**
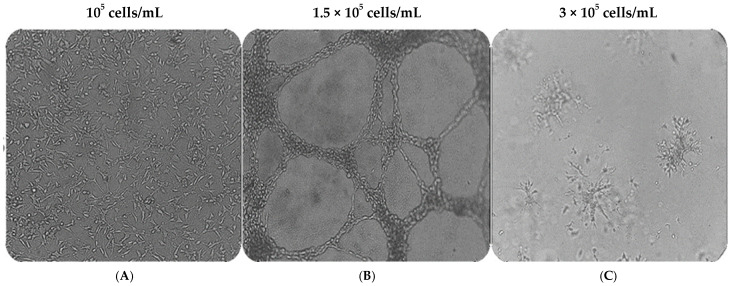
The effect of cell number density on MRC-5 tubulogenesis cultured on Matrigel. MRC-5 cells were seeded in wells at various cell concentrations shown above the panels (**A**–**C**). Note the capillary cord-like structures formed in panel (**B**). Images were captured using a phase contrast microscope at 20× magnification.

To evaluate the ability to undergo angiogenesis, the cord formation experiment is the key method used in vitro. We first tested whether MRC-5 cells can form cord-like networks on the three-dimensional Matrigel matrix, and then we determined the optimal starting cell density needed to establish a “vascular mimicry meshwork” in vitro. For this purpose, cells were seeded on Matrigel using different starting cell densities of 10^5^ cells/mL, 1.5 × 10^5^ cells/mL, or 3 × 10^5^ cells/mL, respectively. Interestingly, cell number has a profound impact on tube formation. MRC-5 cells formed well-defined, distinct interconnected cord-like structures when the seeding concentration was 1.5 × 10^5^ cells/mL (Figure 2A–C). This “vessel-like” network resembles the early stages of endothelial cell tubulogenesis. When cultured under the same conditions but in a lower seeding density (10^5^ cells/mL), MRC-5 cells failed to form any consistent structures, and only small branches, but no tubes, were reported because of the insufficient number of cells seeded on the matrix. As the number of seeded cells increases, the network of tubes expands. However, at higher concentrations (3 × 10^5^ cells/mL), the cells begin to invade the Matrigel matrix and form aggregates.

### 3.2. In Vitro Tube Formation Reveals Different Morphological Patterns

**Figure 3:**
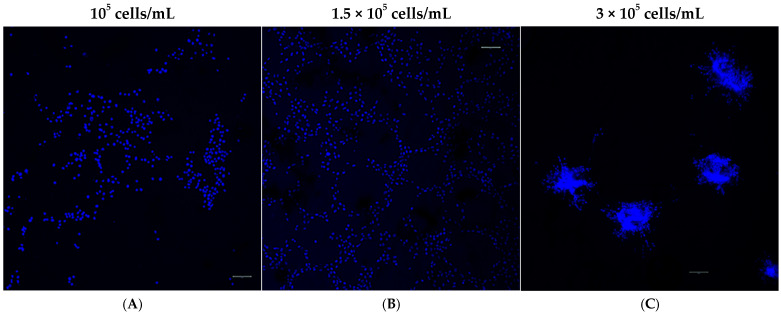
The morphological pattern in MRC-5 cultures seeded on Matrigel at various starting cell densities. (Scale bar = 20 μm). MRC-5 cells were incubated on Matrigel matrix and then fixed and stained with DAPI (blue) for visualizing the nuclei. Samples were visualized under CLSM. The different cell concentrations applied are shown above the panels (**A**–**C**). Abbreviations: CLSM, confocal laser scanning microscopy; DAPI, 4′,6-diamidino-2-phenylindole. Images were captured using a phase contrast microscope at 40× magnification.

To further assess the morphological pattern of MRC-5 cells seeded on Matrigel matrix in various starting densities, using three-dimensional (3D) images, we utilized a confocal laser scanning microscope. The samples were stained with DAPI to label the nuclei of the MRC-5 cells as described in Section 2. Interestingly, cells showed distinct morphological patterns of tubulogenesis in vitro. Specifically, alterations were detected when different starting concentrations of MRC-5 cells were seeded on top of the Matrigel matrix. The results obtained thus far confirmed that cell concentration affects the ability for tube formation (Figure 3A–C). Tubes were poorly formed when the starting cell seeding concentration was too low (10^5^ cells/mL), confirming that this number of cells was not sufficient to initiate capillary structures in vitro. As more cells were seeded (1.5 × 10^5^ cells/mL), a tubal network was formed on top of the Matrigel, and MRC-5 cells demonstrated a growth pattern similar to ECs after 24 h. However, when the cell concentration was raised to 3 × 10^5^ cells/mL, regular cord-like structure formation was not detected. In contrast, the cells became confluent, invaded Matrigel, and initiated the formation of sprouts originating from the previously formed spheroid-like aggregates embedded in the Matrigel.

### 3.3. MRC-5 Cell Cord Formation over Time

To evaluate the cell cord formation of MRC-5 cells over time, a Matrigel-based tube-forming assay was employed for 48 h. Following the assay, representative images of the capillary-like cord structures were taken and subsequent quantitative analysis, recording anastomotic network parameters, using the ImageJ Angiogenesis Analyzer tool [41]. The tube formation parameters analyzed, by using the Angiogenesis Analyzer plugin, include the number of junctions (branching capillary nodes), segments (capillaries delimited by two junctions), and meshes (areas enclosed by segments).

**Figure 4:**
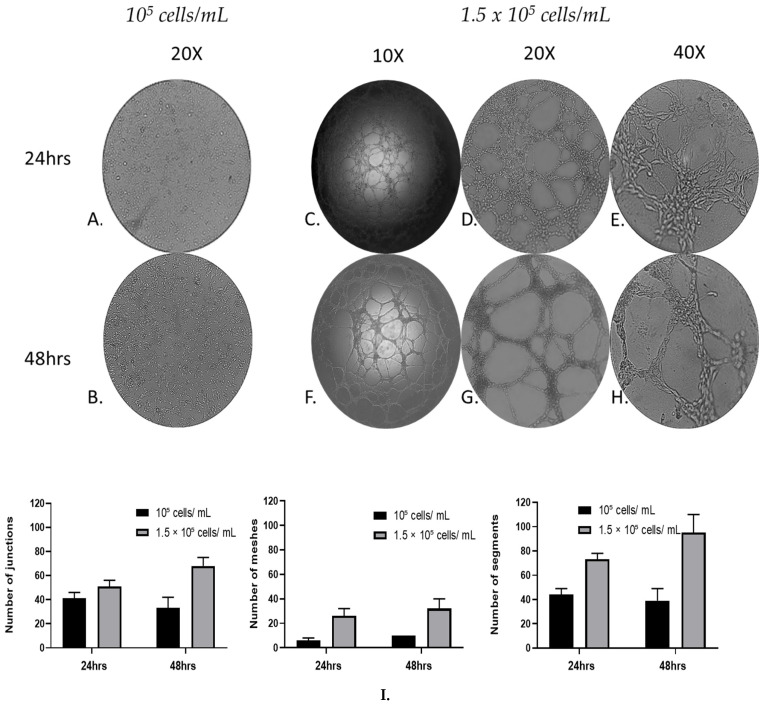
Qualitative and quantitative analysis of MRC-5 cells in vitro tube formation within 48 h. MRC-5 lung fibroblast cells 10^5^ cells/mL (**A**,**B**) and 1.5 × 10^5^ cells/mL were incubated with Matrigel, and the tube formation was recorded at 24 (**A**,**C**–**E**) and 48 h (**B**,**F**–**H**), respectively, under an inverted phase microscope. (**I**) Quantification of the capillary network by Angiogenesis Analyzer plugin. A starting cell density of 1.5 × 10^5^ cells/mL produced consistent mature looping patterns in Matrigel for at least 48 h. Data are representative of measures obtained from six to nine fields.

After 24 and 48 h, the tube-forming assay (Figure 4C–H) revealed that the cultures of MRC-5 cells with a starting seeding concentration of 1.5 × 10^5^ cells/mL formed a complex anastomosis network on the surface of the Matrigel. Interestingly, although in these cultures a network of interconnected capillaries, complex and branched, was formed in the cultures with a starting seeding concentration of 10^5^ cells/mL, the cells did not form capillary-like cord structures (Figure 4A,B). Moreover, the capillary-like cord structure was formed more densely, in a time-dependent manner; the complexity of the network formed by 48 h was higher than that of 24 h in terms of the number of junctions, segments, and meshes. MRC-5 cells gradually formed capillary-like cord structures, and the capillary-like tubes connected to each other created a mesh-like structure on the gel.

### 3.4. Cell Viability and Proliferation

**Figure 5:**
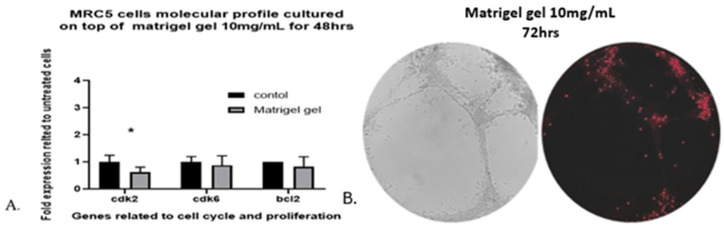
(**A**). qPCR analysis of MRC-5 seeded on top of 10 mg/mL Matrigel matrix gel. The gene expression profiles for proliferation- and cell cycle-related molecules in MRC-5 cells that had been cultured on top of Matrigel matrix gel for 48 h in comparison to untreated MRC-5 control cultures, grown in plates without Matrigel, are presented. The analysis was carried out with qPCR with the primers shown in Supplementary Table S1, as described in Section 2. The data shown above provide evidence of a representative experiment in which three measurements were made to calculate the mean (±SD). A biological replication of this experiment was carried out at least twice with similar results. Statistics were analyzed using a paired *t*-test. A significance level of * *p* < 0.05 denotes significance. (**B**). Propidium iodide (red) staining of human lung fibroblasts in Matrigel hydrogel constructs on day 3 (72 h). Red spots indicate dead cells. The percentile of viability of MRC-5 cells measured by Angiogenesis Analyzer was 18.55%. Images were captured using a phase contrast microscope at 40× magnification.

After 48 h, the viability of human lung fibroblasts cultured on top of Matrigel reached 98.13% of that of untreated MRC-5 cells grown on plates without Matrigel (the control), and the death percentage was less than 4%, as estimated by Image-J software. The results revealed that Matrigel matrix hydrogel had no effect on the viability of MRC-5 cells. As shown in (Figure 4C–H), the capillary-like tubular structure was formed more densely, and the cord structures increased on day 2 (48 h). In addition, the gene expression profiling of three genes related to cell cycle and proliferation, when MRC-5 cells were seeded on top of 10 mg/mL Matrigel matrix hydrogel for 48 h, does not reveal any major effects for two of the three genes tested. The three cell cycle- and proliferation-related genes that were tested with qPCR are Cdk2, Cdk6, and Bcl-2. The reason for selecting only these three genes was that in a previous work, it was shown that only a limited number of proliferation- and apoptosis-related genes can be detected by molecular analysis in MRC-5 cells [36]. Although no significant differences were observed in the levels of Cdk6 and Bcl-2 genes compared to the untreated MRC-5 control cultures, the Cdk2 level was significantly decreased (Figure 5A). This decrease, however, in the gene expression profile of Cdk2 on day 2 (48 h) implies an interruption in the regulation of the cell cycle, a fact that might contribute to proliferation restriction and facilitation of cell structure formation.

To further investigate the viability of MRC-5 cells remaining after 48 h, we used fluorescent dead staining to determine dead cells in the 3D cell-laden constructs, according to the manufacturer’s instructions. By 72 h, the estimated death percentage by Image-J software was 18.55% (Figure 5B). Many branches remained, but many of the cells became apoptotic, and some tubes began to disconnect.

### 3.5. Capillary Sprouting from Gel-Embedded Spheroid-like Aggregates

**Figure 6:**
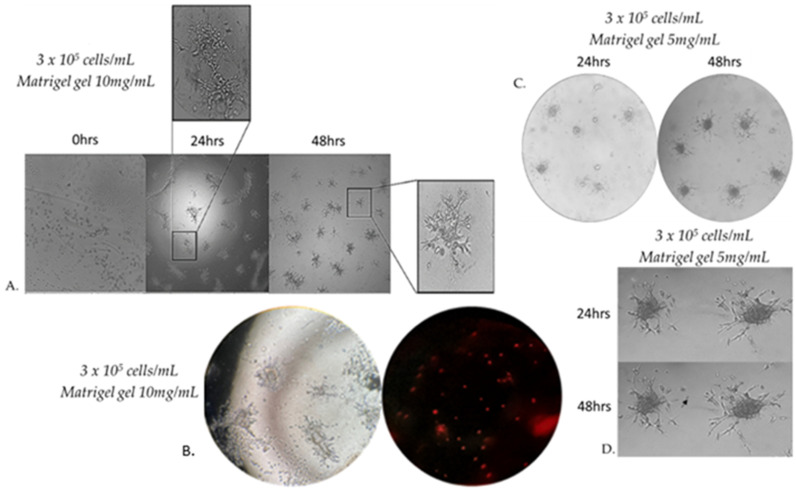
Morphology of capillary sprouts originating from MRC-5 cell spheroid-like aggregates embedded in 10 mg/mL Matrigel matrix gel. Note that gel-embedded spheroid-like aggregates give rise to radially outgrowing capillary sprouts. Outgrowing sprouts of neighboring spheroids grow directionally towards each other to establish networks of anastomosing capillary-like structures, as shown by phase-contrast microscopic analysis (**A**). Capillary-like structures of varying sizes form a true lumen throughout the gel. Cells that are not integrated into the monolayer become apoptotic (**B**). Directional sprouting of capillary-like structures towards each other, originating from two neighboring spheroids, embedded in 5 mg/mL Matrigel gel. Capillary sprouts start to change their direction to grow towards each other (**C**). Note that the centers of the two spheroid-like aggregates have moved closer together after 24 h (arrow) (**D**). Images were captured using a phase contrast microscope at 20× magnification (**A**,**C**) and 40× magnification (**B**,**D**).

When seeding MRC-5 cells at different densities on 10 mg/mL Matrigel gel, we observed that beyond a critical density, cells invaded the matrix and formed aggregates. Cells originating from these formed 3D spheroid-like aggregates that invaded through the gel and gave rise to radially outgrowing capillary sprouts, which subsequently gave rise to complex anastomosing capillary-like networks (Figure 6A). Cells that were not integrated into the monolayer became apoptotic (Figure 6B). The spheroid-like aggregates embedded in 10 mg/mL Matrigel gel were cultured for at least 48 h after their formation. This observation prompted us to more systematically study MRC-5 sprouting in a 3D Matrigel matrix. Since cell adhesion is known to play an important role in the sprouting of cells in 3D matrices, we investigated the effects of the physical properties of the 3D matrix on cell sprouting patterns. We compared cell sprouting in the matrix as a function of matrix density obtained through two different Matrigel concentrations: 5 and 10 mg/mL. As a result, we next extended our studies in an assay in which MRC-5 cells were seeded on 5 mg/mL Matrigel gel (Figure 6C). The outgrowth of these capillary-like structures was assessed qualitatively and quantitatively.

Within a few hours after the formation of the spheroid-like aggregates in 5 mg/mL Matrigel gel, MRC-5 cells originating from the embedded spheroids progressively invaded the Matrigel matrix to form complex networks of capillary-like structures. Before 48 h, some sprouts changed their direction to grow towards a neighboring spheroid-like aggregate if this spheroid is in close proximity (Figure 6C,D).

**Figure 7:**
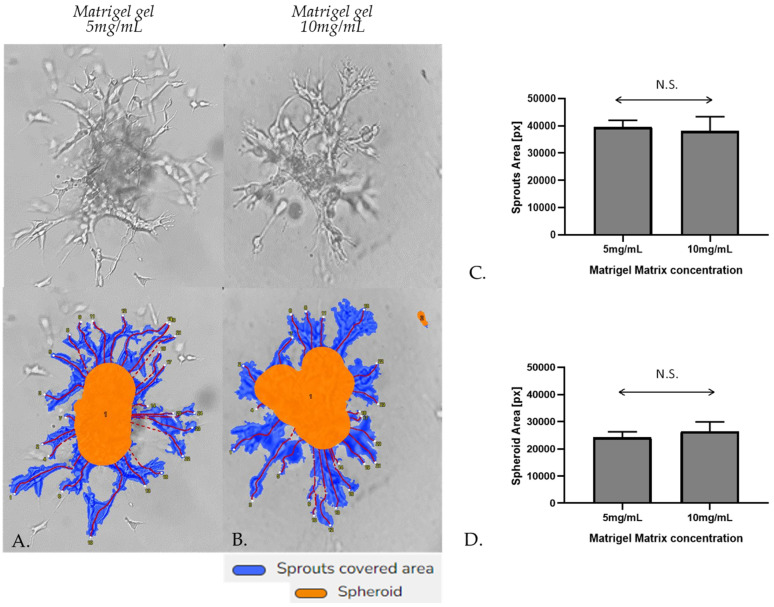
Representative sprouting morphology in 5 mg/mL (**A**) and 10 mg/mL Matrigel (**B**). Upper panel: phase-contrast microscopic images of spheroid-like aggregates. Lower panel: computer-processed images by WimSprout image analysis for sprouting determination (N.S.: not significant). (**C**,**D**) Quantification of sprout outgrowth and spheroid area in 5 mg/mL and 10 mg/mL Matrigel, respectively, using WimSprout analysis. In the computer-processed images, the red line outlines the detected sprouts, and the numbers correspond to individual sprouts identified by the software for quantitative analysis. Images were captured using a phase contrast microscope at 40× magnification.

Embedded spheroid-like aggregates consist of two distinct populations: a migratory or invasive population and a non-migratory core. This is a consequence of the two distinct microenvironments present. Cell–matrix interactions dominate the invasive population and cell–cell contacts dominate the non-migratory core [42]. Initially, the spheroidal cells appeared as a compact structure; however, subsequent cell invasion caused spheroid-like aggregate expansion and resulted in breaches in the spheroid core. The spheroids were photographed after 48 h of incubation by a phase-contrast microscope. Sprout and spheroid area, on matrices of both densities, were quantified by WimSprout (Version 2022.2, Wimasis Image Analysis, Onimagin Technologies SCA, Córdoba, Spain). However, we did not notice any significant difference between 5 mg/mL and 10 mg/mL Matrigel gel (Figure 7A–D).

### 3.6. MRC-5 Cells Invade Through Matrigel Gel

**Figure 8:**
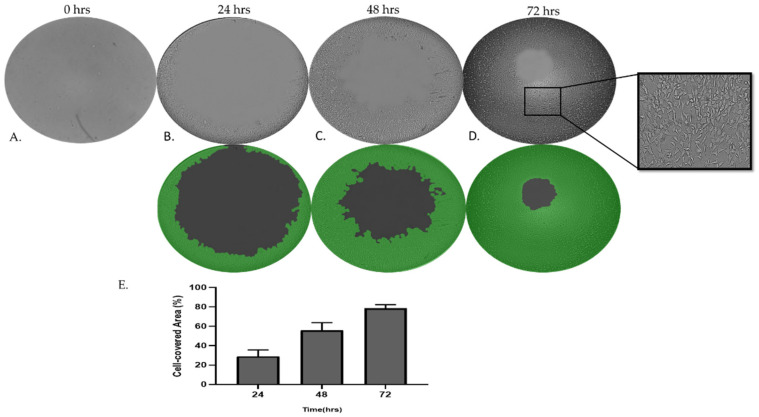
Assay for MRC-5 cell invasion through Matrigel™ using Oris Universal Cell Migration Assembly kit. MRC-5 cells were allowed to invade for 24–72 h through Matrigel^®^ (**A**–**D**). Wells without a Cell Seeding Stopper served as the positive control. (**E**) Quantitative analysis of invasion area using Wimasis image analysis. In the computer-processed images, different colors indicate the segmentation of invaded areas and non-invaded regions used for quantification. Images were captured using a phase contrast microscope at 4× magnification.

To further examine and decouple the invasion capacities of MRC-5 cells on Matrigel matrix gel, we utilized the Oris Universal Cell Migration Assembly Kit as described in Section 2. As we have observed (Figure 8), MRC-5 cells on Matrigel beyond a critical density invade through the gel. Photographs and quantitative analysis of the results revealed that the invasion rate of cells cultured in semi-solid Matrigel increased in a time-dependent manner.

### 3.7. Assessment of Endothelial Biomarkers in the Matrigel-Grown MRC-5 Cell Cultures

**Figure 9:**
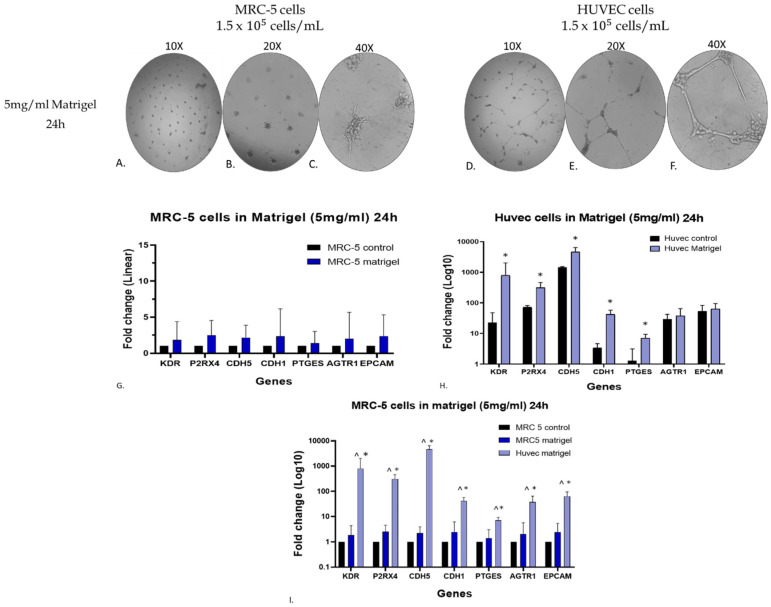
Assessment of gene expression level of endothelial biomarkers by qPCR analysis. MRC-5 (**A**–**C**) and HUVEC (**D**–**F**) cells were grown on Matrigel (5 mg/mL) for 24 h, whereas control cultures were used MRC-5 and/or HUVEC cells grown in plates without Matrigel. Due to the different levels of expression between MRC-5 and HUVEC cells, the expression level of each gene is depicted either as linear fold increase for MRC-5 (**G**) or logarithmic fold change for HUVEC (**H**) +/− standard deviation (n = 3–5). Notably, these obtained gene expression data obtained from MRC-5 and HUVEC cells are also shown in (**I**) to allow the comparison between the two cell lines. The ∗ symbolizes the statistically significant differences in the expression level of each gene between the control group and the Matrigel-grown cell cultures, whereas the ^ indicates statistically significant differences between MRC-5 and HUVEC cells grown on Matrigel.

**Figure 10:**
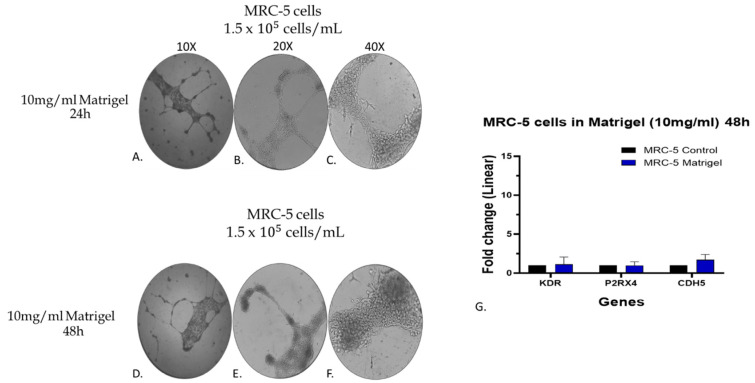
Assessment of the gene expression level of KDR, P2RX4, and CDH5 endothelial biomarkers by qPCR analysis in MRC-5 cells grown on 10 mg/mL Matrigel. The morphology of cells grown for 24 (**A**–**C**) and/or 48 h (**D**–**F**) is shown. MRC-5 cells grown in plates without Matrigel were used as a control culture. In (**G**), the expression level of each gene is depicted as fold change +/− standard deviation (n = 3–5). Images were captured using a phase contrast microscope at 10× magnification for (**A**,**D**), 20× magnification for (**B**,**E**), and 40× magnification for (**C**,**F**).

To further verify at the molecular level, the macroscopic observation of endothelial-like tube formation of MRC-5 cells grown on Matrigel, additional experiments were carried out to assess the gene expression level of seven specific endothelial biomarker genes. The selected genes were Kinase Insert Domain Receptor (KDR), Purinergic Receptor P2X 4 (P2RX4), Cadherin 5 (CDH5), Cadherin 1 (CDH1), Epithelial Cellular Adhesion Molecule (EPCAM), Angiotensin II Receptor Type 1 (AGTR1), and Prostaglandin E Synthase (PTGES) [43]. Notably, we included in this experimentation the well-established endothelial model of the human umbilical vein endothelial cell line (HUVEC cells) to ensure the obtained data [27,37,43]. Initially, the MRC-5 and HUVEC cells (1.5 × 10^−5^ cells/mL) were plated on 5 mg/mL Matrigel and allowed to grow in culture for 24 h. As expected, HUVEC cells displayed canonical tube-forming behavior (Figure 9D–F), while MRC-5 cells formed branched, cord-like structures (Figure 9A–C), distinct from typical endothelial tubes. Also, cultures of MRC-5 and HUVEC cells grown in 24-well plates without Matrigel served as a control for comparative evaluation of the data. As shown in Figure 9H, the gene expression level of all seven endothelial biomarkers studied in Matrigel-plated HUVEC cells showed a statistically significant increase after 24 h incubation, as expected [27]. MRC-5 cells cultured on Matrigel also exhibited upregulation of the same markers (Figure 9G), albeit at levels substantially lower than those observed in HUVECs (Figure 9I). By further evaluating the data taken from MRC-5 cells grown on Matrigel, it is obvious that out of the seven endothelial biomarkers studied, three of them, KDR, P2RX4, and CDH5, presented a higher increase in their gene expression. Based on this observation, an additional and complementary experiment was carried out in which the MRC-5 cell cultures were allowed to grow on a higher Matrigel concentration (10 mg/mL) for a 24–48 h period in cultures before assessing again the gene expression level of these biomarkers. According to the results indicated in Figure 10G, the expression levels of KDR, P2RX4, and CDH5 genes did not exhibit substantial alterations following a 48 h incubation with 10 mg/mL Matrigel compared to the changes seen in MRC-5 cells plated on 5 mg/mL Matrigel (Figure 9G). However, among the three genes, the expression level of CDH5 has shown a higher increase in gene expression (Figure 10G). Noticeably again, MRC-5 cells grown on the higher Matrigel concentration (10 mg/mL) now exhibited the cord-formation structures even from the 24 h period of incubation. In general, these aforementioned data suggest that culture on Matrigel causes limited upregulation of some endothelial-related genes, especially KDR, P2RX4, and CDH5, in MRC-5, although not near the expression levels seen in HUVECs, nor do they represent a complete endothelial transcriptional program. Since there is no expression of canonical endothelial markers and no protein-level validation, these results could best be interpreted as a sign of phenotypic plasticity rather than a conversion into a different lineage. Further work is needed to investigate any potential clinical exploitation of such observations within the concept of regenerative medicine.

### 3.8. RNASeq, Gene Ontology, and Pathway Enrichment Analysis of Differentially Expressed Genes in MRC-5 Cells Expanded in Matrigel

To further understand the molecular mechanisms underlying the observed phenotypic changes in MRC-5 cells cultured on Matrigel, Gene Ontology (GO) and Reactome pathway enrichment analyses were performed on the differentially expressed genes (DEGs) obtained by comparing Matrigel-expanded MRC-5 cells to untreated controls. To elucidate the molecular mechanisms underlying the phenotypic changes observed in MRC-5 fibroblasts cultured on Matrigel, two independent transcriptomic experiments were undertaken, modifying only the duration of Matrigel-based cell culture. To ensure experimental consistency, no modifications were applied to the cell passage number and Matrigel concentration, as the primary objective was to assess alterations in the total transcriptomic landscape and gene-expression profile of the MRC-5 cells induced by prolonged culture conditions. Specifically, the Matrigel-based cultivation period was extended from 24 h to 48 h to detect time-dependent gene expression dynamics affected by the Matrigel microenvironment.

**Figure 11:**
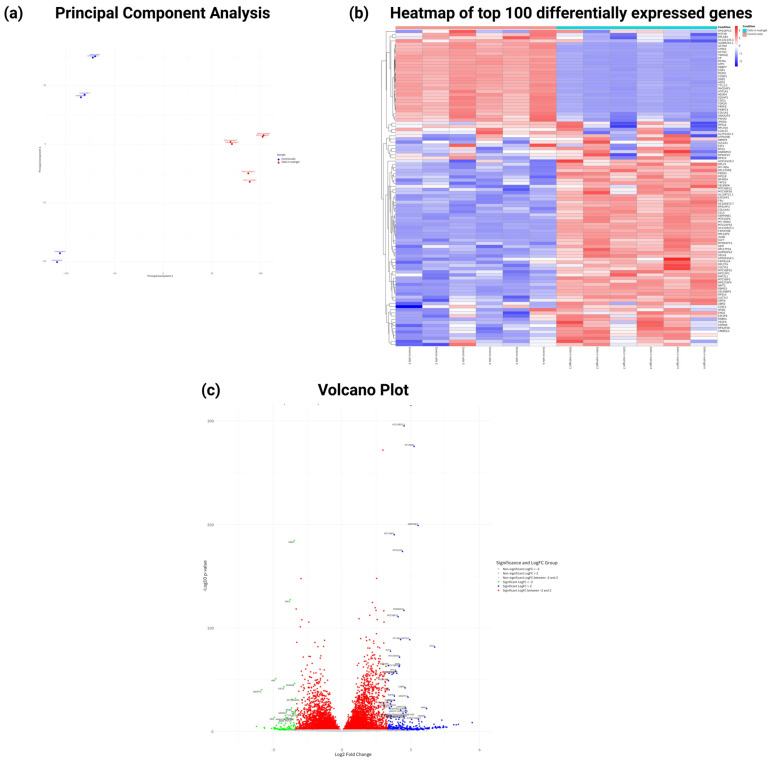
Differential expression gene (DEG) analysis of MRC-5 cells cultured under normal conditions compared to MRC-5 cells embedded in Matrigel for a 24 h duration period. (**a**) Principal component analysis (PCA) plot of gene expression data. (**b**) Heatmap of unsupervised hierarchical clustering of the top 100 deregulated genes based on *p*-value (MRC-5 cells cultured in normal conditions compared to MRC-5 cells cultured in Matrigel) detected by total RNA sequencing. (**c**) Volcano plot displaying the differentially expressed genes between MRC-5 cells cultured in normal conditions and MRC-5 cells cultured in Matrigel. Genes with |logFC| > 1 and *p*-value < 0.05 are considered statistically significant. Only the genes with |logFC| > 2 are labeled in the plot, and the statistically significant genes (*p*-value < 0.05) are colored. Each color corresponds to a different lipid category, which is shown to the right of the plot.

In the 24 h experiment, the design was to carry out differential analysis of gene expression comparing MRC-5 cells cultured under normal conditions with those embedded in Matrigel. The results were obtained using all three biological replicates as both the quantity and quality of the obtained sequencing reads met the required standards. The PCA plot revealed a clear separation between the two conditions tested (Figure 11a). Moreover, unsupervised hierarchical clustering analysis of the top 100 differentially expressed genes validated the expected sample clustering in correspondence with the culture conditions (Figure 11b). Also, the volcano plot shows a large number of both upregulated and downregulated genes between the two groups (Figure 11c).

**Figure 12:**
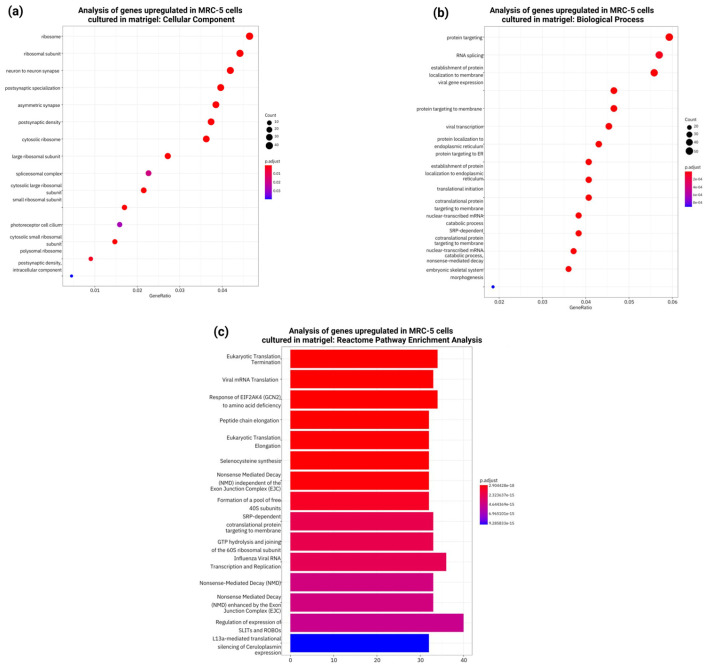
Gene Ontology (GO) enrichment analysis of the 1179 genes significantly upregulated in MRC-5 cells cultured in normal conditions compared to MRC-5 cells cultured in Matrigel for a 24 h duration period. (**a**) Top 15 significantly enriched GO cellular component (CC) terms associated with the upregulated genes in MRC-5 cells cultured in normal conditions compared to MRC-5 cells cultured in Matrigel. The gene ratio and statistical significance (*p*-value < 0.05, following Benjamini and Hochberg’s adjustment method) are also depicted. (**b**) Top 15 significantly enriched GO biological process (BP) terms associated with the upregulated genes in MRC-5 cells cultured in normal conditions compared to MRC-5 cells cultured in Matrigel. The gene ratio and statistical significance (*p*-value < 0.05, following Benjamini and Hochberg’s adjustment method) are also depicted. (**c**) The REACTOME pathway enrichment analysis on the upregulated genes in MRC-5 cells cultured in normal conditions compared to MRC-5 cells cultured in Matrigel. The top 15 statistically significant pathways are listed, and their colors correspond to the adjusted *p*-values.

Continual Gene Ontology enrichment analyses of upregulated genes illustrated consistent significant enrichment with terms associated with nervous system development, synapse-related signaling, and neuronal differentiation (Figure 12a–c). Within these, molecular function domains were dominated by neurotransmitter receptor activity, whereas cellular components focused on the synapse and structures regarded as neuronal. Reactome pathway analysis pinpointed enrichment within pathways related to neurotransmission again, highlighting the possible inclination toward a neuroectodermal-like transcriptional program (Figure 12c).

**Figure 13:**
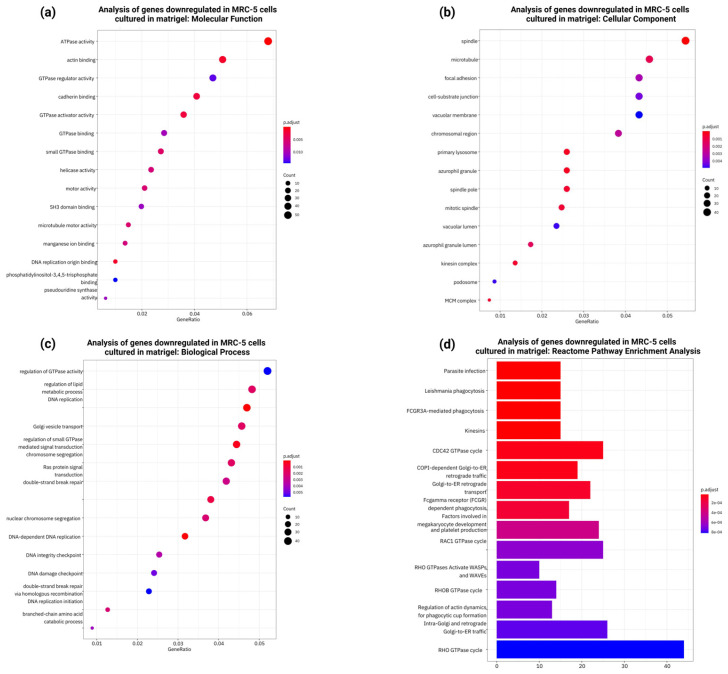
Gene Ontology (GO) enrichment analysis of the 919 genes significantly downregulated in MRC-5 cells cultured in normal conditions compared to MRC-5 cells cultured in Matrigel for a 24 h duration period. (**a**) Top 15 significantly enriched GO molecular function (MF) terms associated with the downregulated genes in MRC-5 cells cultured in normal conditions compared to MRC-5 cells cultured in Matrigel. (**b**) Top 15 significantly enriched GO cellular component (CC) terms associated with the downregulated genes in MRC-5 cells cultured in normal conditions compared to MRC-5 cells cultured in Matrigel. The gene ratio and statistical significance (*p*-value < 0.05, following Benjamini and Hochberg’s adjustment method) are also depicted. (**c**) Top 15 significantly enriched GO biological process (BP) terms associated with the downregulated genes in MRC-5 cells cultured in normal conditions compared to MRC-5 cells cultured in Matrigel. The gene ratio and statistical significance (*p*-value < 0.05, following Benjamini and Hochberg’s adjustment method) are also depicted. (**d**) The REACTOME pathway enrichment analysis on the downregulated genes in MRC-5 cells cultured in normal conditions compared to MRC-5 cells cultured in Matrigel. The top 15 statistically significant pathways are listed, and their colors correspond to the adjusted *p*-values.

Similarly, the analysis performed on the downregulated genes showed enriched GO associated with extracellular matrix refining, cell adhesion, and components of the cytoskeleton (Figure 13a,b). Pathway enrichment of Reactome illustrated the downregulation of collagen biosynthesis pathways, interactions with integrins, and processes related to the remodeling of the matrix (Figure 13c,d).

**Figure 14:**
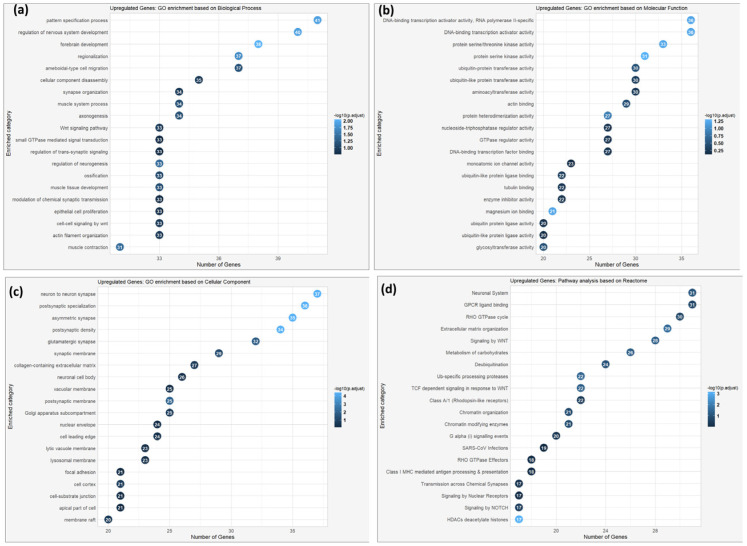
Gene Ontology enrichment analysis based on biological process (**a**), molecular function (**b**), cellular component (**c**), and pathway enrichment analysis (**d**) based on the Reactome database of the upregulated genes of MRC-5 cells expanded in Matrigel for a 48 h duration period. The number of genes enriched within each GO term and the statistical significance (*p*-value < 0.05, following Benjamini and Hochberg’s adjustment method) are also depicted.

For the independent 48 h experiment, another round of RNA-seq analysis was conducted, which again compared MRC-5 cells grown under standard laboratory culture conditions to those embedded in Matrigel for a prolonged duration. One biological replicate of the three in the Matrigel condition was omitted from downstream analysis because of the low sequencing depth and its divergence in clustering in the principal component analysis. So, the results were obtained using two biological replicates per condition, merged with technical replicates to gain more significance in the data. GO enrichment analysis of genes upregulated (log2 fold change >1) in the Matrigel-expanded MRC-5 cells revealed GO terms associated with nervous system development, neuron-to-neuron synapse, and neuronal cell body processes (Figure 14a–c). Molecular functions enriched are neurotransmitter receptor activity and calcium ion binding, while the most enriched cellular components were synaptic membranes and neuronal projections. Such findings were corroborated by Reactome pathway analysis, which revealed the highly significant enrichment of pathways involved in neurotransmission and synaptic signaling (Figure 14d).

**Figure 15:**
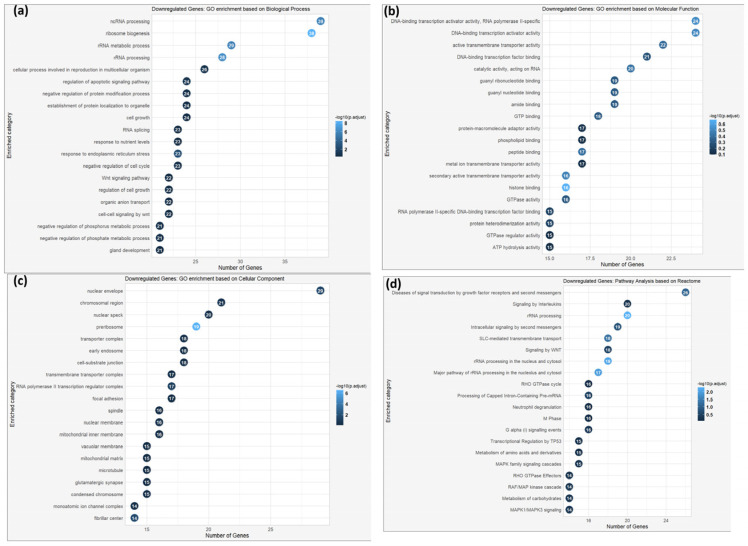
Gene Ontology enrichment analysis based on biological process (**a**), molecular function (**b**), cellular component (**c**), and pathway enrichment analysis (**d**) based on the Reactome database of the downregulated genes of MRC-5 cells expanded in Matrigel for a 48 h duration period. The number of genes enriched within each GO term and the statistical significance (*p*-value < 0.05, following Benjamini and Hochberg’s adjustment method) are also depicted.

Conversely, the downregulated genes (log2 fold change < −1) were majorly involved in extracellular matrix organization, cell adhesion, and cytoskeletal organization (Figure 15a–c). The relative Reactome pathways comprised collagen biosynthesis, integrin signaling, and extracellular matrix–receptor interaction (Figure 15d).

It is noteworthy that, irrespective of the Matrigel-based cultivation duration, both transcriptomic experiments, 24 h and 48 h, performed in parallel reached a unified biological conclusion. Exposure to Matrigel consistently drove transcriptional programs associated with the development of the nervous system, synaptic signaling, and neuronal-like gene expression, devoid of any signs of epithelial or endothelial transitions. Notably, the prolonged cultivation period within the Matrigel induced a more pronounced neuronal-like gene expression profile. However, extended exposure to Matrigel was associated with reduced cell viability, which likely contributed to the decreased concentration of the isolated RNAs and subsequently the quantity of sequencing reads observed in the next-generation sequencing (NGS) analysis. Overall, the data indicate that MRC-5 fibroblasts exhibit some level of neuroectodermal transcriptional plasticity when exposed to Matrigel.

## 4. Discussion

Our initial aim was to investigate the potential of MRC-5 fibroblasts as an alternative cell source for vascular tissue engineering, a field challenged by the limited availability of autologous endothelial cells. The demonstrated ability of these readily available and well-characterized fibroblasts to autonomously self-organize into stable, complex, and neuro-mimetic networks is a significant finding.

The present study aimed to clarify whether human lung FBs can exhibit cord-like morphological and transcriptomic features in response to a three-dimensional extracellular matrix environment. Differentiation was evaluated by the formation of cord-like networks on top of the thick gel of Matrigel and also the alignment of capillary sprouts to form complex three-dimensional networks of potential new vessels. Our data support the hypothesis that MRC-5 cells have the potential to undergo tubulogenesis upon culture on Matrigel and that cell density influences the capacity of tube formation in vitro [44]. These effects likely reflect phenotypic adaptation, possibly linked to their lung origin or neuroectodermal-like transcriptional signatures [45,46,47,48,49], rather than mesenchymal-to-endothelial transition. In fact, the propensity of MRC-5 aggregates to support angiogenesis-like sprouting could be influenced by fibroblast density, although the role of proliferation rate on this phenomenon remains to be explored in future studies. The presented results are in line with similar observations reported for in vivo differentiation of FBs [50]. Nevertheless, enrichment of neuroectodermal and synaptic signaling pathways from transcriptomic analysis cannot secure a conclusive lineage identity. The absence of lineage-specific markers (e.g., surfactant protein-C and chromogranin A) is a limitation; further, Matrigel’s murine endothelioma origin and growth factor composition [51] might bias the outcome. Further studies are thus warranted to ascertain whether MRC-5 fibroblasts can truly acquire lineage-specific features.

While earlier reports indicated fibroblast plasticity toward endothelial lineages under injury or reprogramming conditions [21,22,23,26,28,29,31,52], our analysis revealed that the MRC-5 fibroblasts in Matrigel undergo an alternate transcriptional trajectory enriched in neuronal signatures, possibly mimicking alveolar [48,49] or epithelial structures [18].

Nevertheless, the present study provides novel insights into the morphological and transcriptional plasticity of human lung fibroblasts in a 3D matrix environment. When cultured on top of or embedded within Matrigel, MRC-5 cells formed cord-like structures reminiscent of vascular patterning. While such structures morphologically resemble angiogenic networks, our findings do not support canonical endothelial differentiation. The matrix has previously been used in numerous in vitro and in vivo studies, exhibiting interesting results regarding cell viability, migration, and proliferation [53]. Our results are in line with these previous findings. MRC-5 cells went through several passages before the start of experiments, thus preventing ECs from the original tissue sample from attaching and proliferating. The use of several distinct single-cell clones also greatly reduces the risk of false positives as a result of contamination.

Paracrine effects, such as pleiotropic actions of VEGF-induced sprouting [25,26,27,28,29,30,31], might be considered as an explanation [27,54,55,56,57]; however, our transcriptional evidence weighs in favor of neuroectodermal programs, emphasizing the divergence from those of canonical endothelial differentiation. However, we cannot proceed in characterizing these structures as capillaries of direct endothelial origin in the absence of functional and molecular features.

Transcriptomic data provide further support for these observations. Both independent RNA-seq experiments demonstrated that MRC-5 cells exposed to Matrigel consistently displayed upregulated gene expression programs associated with nervous system development, including synaptic signaling and neurogenesis, rather than endothelial or epithelial gene signatures. These observations align with previous studies that emphasize the importance of the physical and biochemical characteristics of the extracellular matrix in regulating tube formation and cell fate based on spatial and mechanical signals [58].

Interestingly, the GO enrichment analysis of upregulated genes indicated enriched gene terms related to neurotransmitter receptor activity, calcium ion binding, and synaptic structure. While these processes are unrelated to endothelial identity, they may represent an expression of latent developmental plasticity of fibroblasts, or a generic transcriptional response to the neurogenic elements contained in Matrigel, which include laminin, agrin, and nidogen. Given the embryonic origins and mesenchymal capabilities of MRC-5 cells, this gene expression shift may be a manifestation of intrinsic plasticity via a 3D matrix microenvironment. Also, when transcriptomic experiments were conducted previously for MRC-5 cells, it was clear that these cells achieved substantial gene expression reprogramming during replicative senescence and demonstrated their capacity to reprogram their gene expression in relation to variability in the external microenvironment [59]. Additional functional and mechanistic studies are needed to clarify whether these neuronal gene signatures contribute to or merely accompany the endothelial-like transition observed in this system.

Moreover, VEGF, contained in Matrigel, may drive sprouting behavior, as no sprouts are observed in growth factor–deficient conditions, when MRC-5 spheroid-like aggregates are embedded in PuraMatrix Peptide Hydrogel, which is a synthetic matrix used to create defined three-dimensional (3D) microenvironments for a variety of cell culture experiments (Figure S2). However, future studies could complement our findings with detailed analyses of size, viability, and hypoxia-related markers in MRC-5 spheroid-like aggregates. Finally, invasion is generally perceived to be a late event during the progression of human cancer, and previous research has shown that cancer-associated fibroblasts (CAFs) induce invasion of cancer cells through the remodeling of the ECM [60]. However, in the current study, the ability of these specific cells to invade the gel was tested for the first time, not the ability of MRC-5 cells to induce the invasion of cancer cells.

Therefore, it is highly plausible that the MRC-5 cells are responding directly to these neurogenic cues within the matrix, activating a latent transcriptional program that is unrelated to an endothelial fate. This highlights a critical consideration for in vitro studies: the observed cellular phenotype can be profoundly influenced by the specific composition of the biomimetic environment.

Our results indicate that MRC-5 fibroblasts exhibit a robust plasticity in structure and transcription that allows them to form vascular-like, cord-like networks in response to the 3D matrix cues; however, they do not adopt an unequivocal endothelial identity. This ability, which is reported here for the first time, supports knowledge about the plasticity of fibroblasts, and it may have potential implications in tissue engineering and regenerative medicine. Despite the resemblance of these structures to vascular networks, they did not show functional and molecular evidence of differences between non-endothelial and endothelial identity. Furthermore, although MRC-5 fibroblasts may align to form vascular-like cords, whether such cords serve any vascular function was not assessed. Thus, they are not to be considered as functional vasculature. Future work can employ dextran permeability assays or in vivo transplantation models to check whether or not the fibroblast cord has any functional capacity. If so, induced fibroblasts may provide a new cell source to generate vascular-like structures in the engineered tissues in vitro and engineered tissue grafts. The implications of the research will enhance future studies on the therapeutic capability of human fibroblasts, such as MRC-5 cells, which already lend themselves to vaccine development [18,61].

This study provides novel insights into fibroblast plasticity, helping define a clear path for future research. However, the limitations of our study ought to be mentioned. Firstly, we depended on transcriptomic and morphological analysis, and further phenotypic analysis of phenotypic, endothelial, or mesenchymal markers by immunostaining would bolster the plasticity that was observed and would be valuable in confirming the vascular-like structures observed. Furthermore, to deconstruct the specific signals driving the neuroectodermal shift, future experiments must move towards chemically defined synthetic hydrogels where individual ECM components can be added back systematically. This would allow for the precise identification of the molecules responsible for inducing this specific plastic response, building upon our preliminary work with a synthetic matrix. In fact, one key limitation of this work is the lack of validation of changes at the protein level. Although RNA-seq data were supported by targeted qPCR, future work should include immunofluorescence or Western blots for neuronal and endothelial markers (e.g., β-III-tubulin, vWF, CD31) to confirm the observed transcriptional signatures. Secondly, our study did not aim to develop a complex multicellular spheroid model, which has already been widely covered in the literature. Instead, it actually highlighted the unforeseen plasticity of the fibroblast in 3D matrices, an insight that may hold important applications in tissue engineering and stromal cell biology. Thus, while MRC-5 fibroblast aggregates are observed, which are a type of spheroid, a thorough evaluation of their size, viability, and hypoxic status was not conducted. Additionally, although sprouting seemed to be affected by fibroblast density, sprouting proliferation rates were not measured and ought to be researched in the future. Lastly, all the data was obtained in vitro with Matrigel, a growth factor-rich matrix, which may differ from actual physiological conditions. Conducting studies in in vivo models and defined matrices would help in understanding the relevance of fibroblast plasticity.

## 5. Conclusions

The present study investigated whether human lung MRC-5 fibroblasts can undergo morphological and transcriptional plasticity suggestive of vascular-like organization when cultured in a 3D Matrigel matrix. Combining tube formation studies, invasion assays, and transcriptomic profiling, our aim was to establish whether these fibroblasts undergo mesenchymal-to-endothelial transition or instead acquire traits of alternative lineages. Evidence presented in the paper indicates that whereas the MRC-5 fibroblasts did form cord-like networks resembling vascular structures, they did not acquire a clear endothelial transcriptional signature. Instead, the cells expressed gene programs highly enriched for nervous system development and synaptic function, suggesting a Matrigel-induced microenvironment-mediated neuroectodermal-like response. This divergence from the original hypothesis points to a potentially more promising application for fibroblasts in regenerative medicine: not as endothelial substitutes, but as cells capable of organizing themselves into multilayered, neuro-mimetic networks. These properties may prove useful in engineering innervated tissues or living scaffolds that aid neural patterning. Further studies must be undertaken to elucidate the physiological relevance of these fibroblast-derived structures and to elaborate their consequences for treatment modalities.

## Data Availability

The original contributions presented in this study are included in the article/Supplementary Materials. Further inquiries can be directed to the corresponding author.

## Supplementary Materials

The following supporting information can be downloaded at https://www.mdpi.com/article/10.3390/cells14191519/s1. Table S1: Primer sequences used to analyze gene expression profiles by qPCR; Table S2: Primer sequences of the endothelial biomarker genes used in qPCR Analysis; Figure S1: Comparison between MRC-5 and HSC-3 spheroids embedded in Matrigel coated wells. Please note the difference between MRC-5 and HSC-3 cells, since HSC-3 spheroids didn’t give rise to sprouts, for at least 48 h after seeding; Figure S2. MRC-5 cells cultured on top of PuraMatrix Peptide Hydrogel. Please note that tube formation was not observed in growth factor–deficient conditions, when MRC-5 cells were seeded on top of three concentrations (0.25% *w*/*v*, 0.5% *w*/*v* and 1% *w*/*v*) of PuraMatrix Peptide Hydrogel.
