# Supplementary material for: Matrix-Guided Vascular-like Cord Formation by MRC-5 Lung Fibroblasts: Evidence of Structural and Transcriptional Plasticity
Source: Cells. 2025 Sep 29;14(19):1519. doi: 10.3390/cells14191519 (PMC12524158; doi:10.3390/cells14191519)
Supplement: Supplementary file 1 [file cells-14-01519-s001.zip › cells-3792706-supplementary.pdf]

Supplementary Information

Matrix-guided vascular-like cord formation by MRC-5 lung fibroblasts: evidence of structural and transcriptional plasticity

Nikoleta F. Theodoroula[1], Alexandros Giannopoulos-Dimitriou[1], Aikaterini Saiti[1], Aliki Papadimitriou-Tsantarliotou[1], Androulla N. Miliotou[2], Giannis Vatsellas[3], Yiannis Sarigiannis[2], Eleftheria Galatou[2], Christos Petrou[2], Dimitrios G. Fatouros[4], Ioannis S. Vizirianakis[1,2,*]

[1] Laboratory of Pharmacology, School of Pharmacy, Aristotle University of Thessaloniki, Thessaloniki 54124, Greece; theodorn@pharm.auth.gr (N.F.T.); gianalex@auth.gr (A.G-D.); skaikater@auth.gr (A.K.); alikipapadi@pharm.auth.gr (A.P-T.); ivizir@pharm.auth.gr (I.S.V.)

[2] Department Health Sciences, School of Life & Health Sciences, University of Nicosia, Nicosia 2417, Cyprus; miliotou.a@unic.ac.cy (A.N.M); sarigiannis.i@unic.ac.cy (Y.S.); galatou.e@unic.ac.cy (E.G.); petrou.c@unic.ac.cy (C.P.); vizirianakis.i@unic.ac.cy (I.S.V.)

[3] Greek Genome Center, Biomedical Research Foundation Academy of Athens, 115 27 Athens, Greece; gvatsellas@bioacademy.gr (G.V.)

[4] Department of Pharmaceutical Technology, School of Pharmacy, Aristotle University of Thessaloniki, Thessaloniki 54124, Greece; dfatouro@pharm.auth.gr (D.G.F.)

* Correspondence: ivizir@pharm.auth.gr; vizirianakis.i@unic.ac.cy (I.S.V.)

Table S1. Primer sequences used to analyze gene expression profiles by qPCR

HGNC Gene	Primer code	Primer sequence	PCR product size
CDK2 (cdk2)	hCdk2 RT F	5'-TTGTCAAGCTGCTGGATGTC-3'	126bp
	hCdk2 RT R	5'-TGATGAGGGGAAGAGGAATG-3'	
CDK6 (cdk6)	hCdk6 RT F	5'-TGCACAGTGTCACGAACAGA-3'	150bp
	hCdk6 RT R	5'-ACCTCGGAGAAGCTGAAACA-3'	
BCL2 (bcl-2)	hBcl2 RT F	5'-ACTTCGCCGAGATGTCCA-3'	123bp
	hBcl2 RT R	5'-CAAAGAAGGCCACAATCCTC-3'	

Table S2. Primer sequences of the endothelial biomarker genes used in qPCR analysis.

Primer-Name	Primer -Sequence 5' - 3'
Forward CDH1	GCCTCCTGAAAAGAGAGTGGAAG
Reverse CDH1	TGGCAGTGTCTCTCCAAATCCG
Forward EPCAM	GCCAGTGTACTTCAGTTGGTGC
Reverse EPCAM	CCCTTCAGGTTTTGCTCTTCTCC
Forward CDH5	GAAGCCTCTGATTGGCACAGTG
Reverse CDH5	TTTTGTGACTCGGAAGAACTGGC
Forward P2RX4	GTGGCGGATTATGTGATACCAGC
Reverse P2RX4	CACACAGTGGTCGCATCTGGAA
Forward PTGES	GAGGATGCCCTGAGACACGGA
Reverse PTGES	CCAGAAAGGAGTAGACGAAGCC
Forward KDR	GGAACCTCACTATCCGCAGAGT
Reverse KDR	CCAAGTTCGTCTTTTCCTGGGC
Forward AGTR1	CAGCGTCAGTTTCAACCTGTACG
Reverse AGTR1	GCAGGTGACTTTGGCTACAAGC

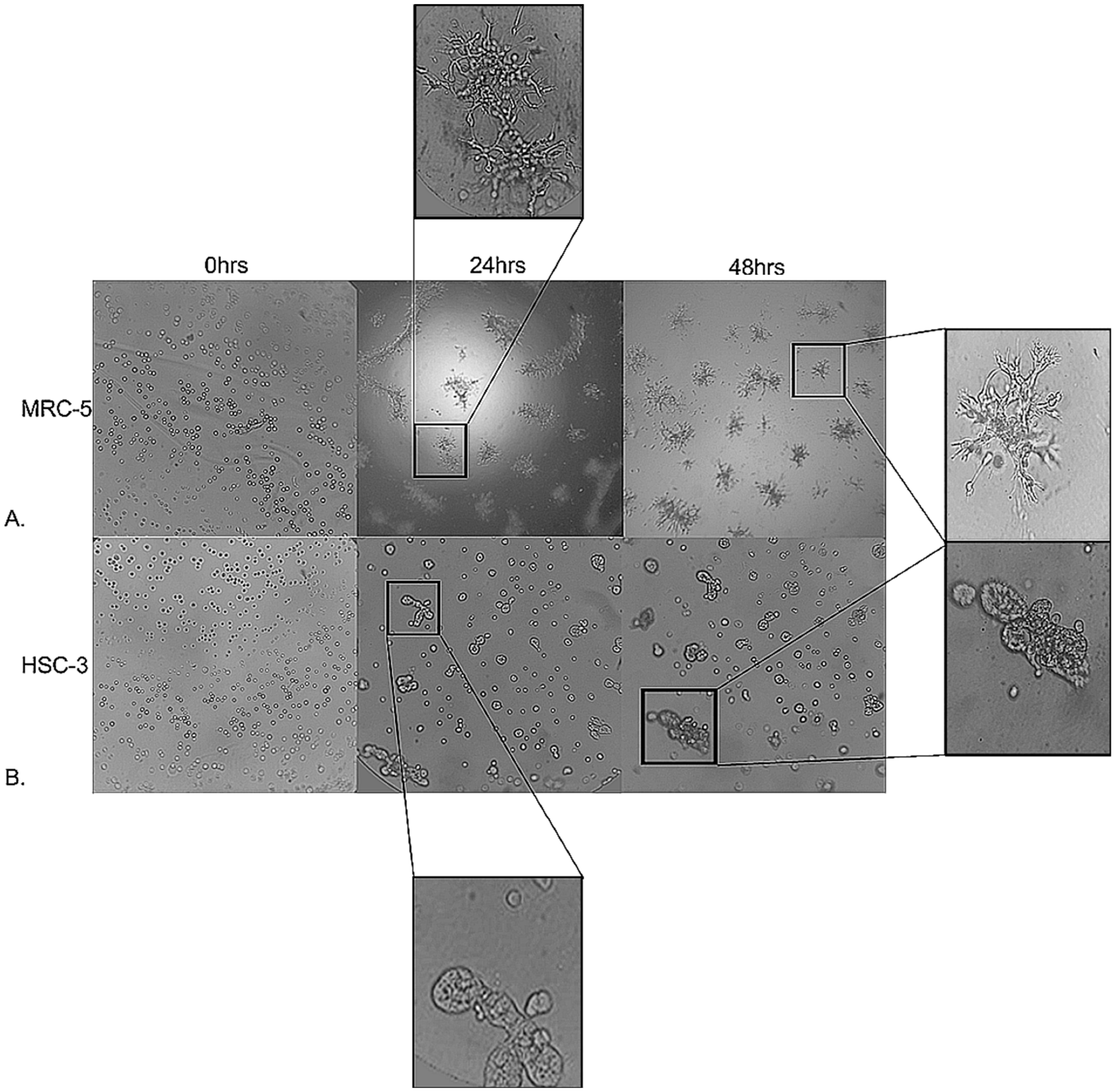

Figure S1. Comparison between MRC-5 and HSC-3 spheroids embedded in Matrigel coated wells. Please note the difference between MRC-5 and HSC-3 cells, since HSC-3 spheroids didn't give rise to sprouts, for at least 48hrs after seeding.

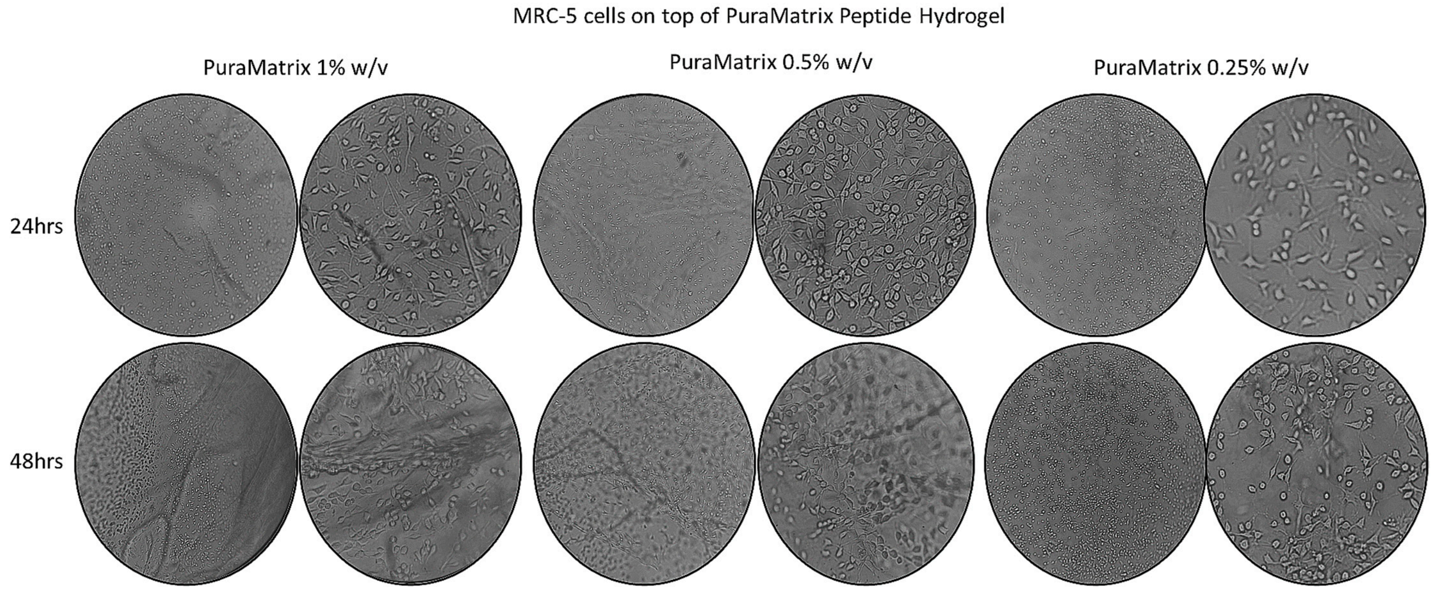

Figure S2. MRC-5 cells cultured on top of PuraMatrix Peptide Hydrogel. Please note that tube formation was not observed in growth factor–deficient conditions, when MRC-5 cells were seeded on top of three concentrations (0.25% w/v, 0.5%w/v and 1% w/v) of PuraMatrix Peptide Hydrogel.
